# Assessment of Prostate MR Image and Predictive Value for Benign Prostate Disease among Different DWI Sequences

**DOI:** 10.2174/0115734056329976241209112720

**Published:** 2025-01-02

**Authors:** Hanli Dan, Lu Yang, Yuchuan Tan, Yipeng Zhang, Yong Tan, Jing Zhang, Min Li, Meng Lin, Jiuquan Zhang

**Affiliations:** 1 Department of Radiology, Chongqing University Cancer Hospital & Chongqing Cancer Institute & Chongqing Cancer Hospital, Chongqing, 400030, China; 2 Department of Radiology, Key Laboratory for Biorheological Science and Technology of Ministry of Education, State and Local Joint Engineering Laboratory for Vascular Implants, Bioengineering College of Chongqing University, Chongqing, 400030, China

**Keywords:** Prostate, Magnetic resonance imaging, Diffusion-weighted imaging, Value, Intra-class correlation coefficient, Receiver operating characteristic

## Abstract

**Background:**

Early diagnosis of prostate cancer can improve the survival rate of patients on the premise of high-quality images. The prerequisite for early diagnosis is high-quality images. ZOOMit is a method for high-resolution, zoomed FOV imaging, allowing diffusion-weighted images with high contrast and resolution in short acquisition times. RESOLVE DWI is an advanced MRI technique developed to obtain high-resolution diffusion-weighted images with reduced susceptibility-related artifacts.

**Objective:**

This study aimed to compare the image quality of conventional single-shot Echo-planar Imaging (ss-EPI) Diffusion-weighted Imaging (DWI), zoomed FOV imaging (ZOOMit) DWI, and readout segmentation of long variable echo-trains (RESOLVE) DWI sequences for prostate imaging, and optimize the strategy to obtain high-quality Magnetic Resonance Imaging (MRI) in order to discriminate malignant and benign prostate diseases.

**Methods:**

Fifty-one patients were enrolled, including 31 with prostate cancer, 11 with prostate benign disease, and 9 with bladder cancer. Patients underwent MRI scans using T2-weighted (T2W), ss-EPI DWI, ZOOMit DWI, and RESOLVE DWI (b = 0, 50, 1400 s/mm^2^) sequences using a 3.0T MRI scanner. Subjective scores of image quality were evaluated by two independent radiologists. Differences in the subjective scores and objective parameters among the three sequences were compared. The agreement and consistency between the findings of the two raters were evaluated with Kappa or Intra-class Correlation Coefficient (ICC). Receiver Operating Characteristic (ROC) curves were used to distinguish malignant and benign prostate disease.

**Results:**

The agreement of subjective scores of 51 patients was high or moderate between the two radiologists (kappa: 0.529–0.880). ZOOMit displayed the highest clarity and the lowest distortion and artifacts compared to ss-EPI and RESOLVE. The two radiologic technicians obtained moderate or high consistency of objective measurement (ICC: 0.527–0.924). In the ROC analysis, ADCmean and Prostate Imaging Reporting and Data System (PI-RADS) scores for three sequences were comparable in differentiating prostate cancer from benign prostate disease (all *p*>0.05), in which ZOOMit indicated the highest Area Under the Curve (AUC) (0.930 and 0.790, respectively).

**Conclusion:**

Compared to the other two sequences, ZOOMit can be deemed preferable to improve prostate MRI diffusion imaging as it has exhibited the highest AUC in identifying prostate cancer.

## 1 INTRODUCTION

Prostate cancer is the second leading cause of malignancy in men [1]. The incidence of prostate cancer is increasing gradually; hence, an early diagnosis is important, especially for improving the quality of life and 5-year survival rate of patients.

Currently, multiparametric Magnetic Resonance Imaging (mpMRI) is one of the most effective imaging methods for the diagnosis of prostate cancer. Recent studies have shown that mpMRI is useful in differentiating organ-confined prostate cancer from advanced prostate cancer and that pre-biopsy mpMRI is valuable in ruling out insignificant prostate cancers, consequently reducing the number of patients who require a biopsy [2, 3]. Despite its advantages, mpMRI has some limitations. One main disadvantage of mpMRI is the lack of standardization. Diffusion-weighted Imaging (DWI) for prostate imaging is applied using various methods and different b factors across different institutions, as a result of which the reported ADC values for prostate cancer differ considerably. Currently, mpMRI is one of the most effective imaging methods for the diagnosis of prostate cancer. DWI for prostate imaging is applied using various methods and different b factors; hence, the reproducibility of mpMRI results is scarce. The imaging distortions and susceptibility artefacts are other problems in using DWI, which may pose challenges in assessing the therapeutic response. Wong OL *et al*. found that MR-Linac DWI scans exhibited significantly more severe geometric distortion than MR-simulator scans (*p* < 0.01), and most phantom measurements fell within the image in-plane resolution of 3 mm [4].

In recent years, various novel MRI techniques have been developed. One such technology uses the DWI sequence, which has been preferred by various researchers and clinical professionals because of its advantages of high diagnostic accuracy, operational convenience, and image diversity [5]. The prostate cancer cells are densely arranged; therefore, the movement of water molecules is restricted, which leads to increased signals in DWI images and a clearer identification of the structures [6-8]. The ZOOMit DWI technique has been demonstrated to be suitable for both DWI and Diffusion Tensor Imaging (DTI) of the prostate [9, 10]. Several previous studies have demonstrated that the Apparent Diffusion Coefficient (ADC) map and high b-value images could better display the anatomic structures and reduce false images, double-image signals, and distortions [11]. The RESOLVE DWI sequence also showed short echo spacing when scanning the head, neck, and breast. These images were less distorted with a clear display of anatomic details. Therefore, the RESOLVE technique could provide high-quality images [10, 12, 13]. These new technologies have facilitated better-quality prostate images and an improved ability to distinguish between benign and malignant tissues. Thus far, no studies have compared the use of ss-EPI, ZOOMit, and RESOLVE sequences in prostate disease diagnosis.

The PI-RADS scoring system, released by the American Society of Radiology, is mainly based on subjective scoring of multi-parameter MR (T2WI, DWI, and dynamic enhanced sequence images) and is used to detect clinically significant cancers. It is a bridge for communication between radiologists and urologists.

Therefore, here, we aimed to compare the image quality of prostate MRI using ss-EPI DWI, ZOOMit DWI, and RESOLVE DWI, and further compared the ADC and PI-RADS scores to differentiate prostate cancer from benign prostate disease, which could optimize the strategies for better accuracy of MRI diagnosis of the prostate diseases.

## 2 MATERIALS AND METHODS

### 2.1 Patients

This study was approved by the ethics committee of Chongqing University Cancer Hospital (approval ID: CZLS2020248-A). The informed consent of the patients was waived by the ethics committee of Chongqing University Cancer Hospital. In this study, we retrospectively analyzed the data of 51 patients, including 31 with prostate cancers, 11 with benign lesions, and 9 with bladder cancers, who were outpatients and inpatients at our hospital and underwent prostate MRI between April 2020 and August 2020. The age ranged from 48 to 85, with the median being 68. Inclusion criteria were as follows: (1) patients who underwent prostate MRI in our hospital; (2) none of the patients received prostate puncture within 4-6 weeks before undergoing MRI. The exclusion criteria were as follows: (1) patients with pelvic bone surgery (metal artifacts); (2) patients having incomplete diffusion sequences.

### 2.2 Imaging Parameters

Patients underwent MRI scans using T2-weighted (T2W), ss-EPI DWI, ZOOMit DWI, and RESOLVE DWI (b = 0, 50, 1400 s/mm^2^) sequences using a 3.0T MRI scanner (MAGNETOM Prisma, Siemens Healthcare, Erlangen, Germany) and an eighteen-channel phased-array body coil. Imaging parameter data are presented in Table **1**.

### 2.3 Subjective Image Quality Evaluation

We invited two radiologists (Lu Yang and Hanli Dan), having 12 and 8 years of experience in the urinary system, respectively, to provide subjective evaluation of prostate images. To avoid reading bias, the images acquired using ss-EPI DWI and RESOLVE DWI sequences were magnified to the size of those obtained using ZOOMit DWI sequences. The images were subjectively evaluated by the two readers side by side so that they were blinded to the scores rated by each other. Previously, the Likert scale was adopted by Hellms *et al*. and Brendle *et al*. for the subjective evaluation of prostate images [13, 14]. Therefore, the radiologists evaluated the image clarity, visual distortion, and artifacts in all DWI images, and classified the image quality according to the Likert scale. Using this scale, image quality was rated on a scale of 1-4 as follows: 1 = poor image clarity/severe distortion or artifacts; 2 = moderate image clarity/moderate distortion or artifacts; 3 = good image clarity/slight distortion or artifacts; and 4 = excellent image clarity/no distortion or artifacts. According to PI-RADS version 2.1, the two radiologists completed PI-RADS scores for all 3 DWI sequences. They were blinded to either the pathology results or the results evaluated by the other radiologist.

### 2.4 Objective Image Quality Evaluation

The Signal-to-noise Ratio (SNR) and Contrast-to-noise Ratio (CNR) analyses were conducted by two radiologic technicians with 12 and 3 years of experience. The Regions of Interest (ROIs) were defined manually on images acquired through T2W and DWI sequences at b = 50, 1400 s/mm^2^. The SNR was defined as the signal intensity of the entire prostate divided by the Standard Deviation (SD). Signal Intensity (SI) in corresponding areas of the obturator internus muscle was measured. The SNR and CNR were calculated as follows:


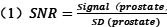



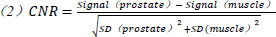


The two radiologic technicians manually measured the Right-left (RL) [14] diameter, Anterior-posterior (AP) diameter, ratio of Anterior-posterior diameter/Right-left diameter (AP/RL), and area of the prostate from the acquired images. The outline of the prostate on the cross-sectional T2W TSE image was used as the anatomic shape of the prostate, and then, all three DWI images were compared with the T2W images. Two radiologic technicians delineated the lesion area of the prostate as ROIs and measured the ADC value of the lesion in all 3 DWI sequences of patients with prostate benign disease or cancer [15, 16].

### 2.5 Statistical Analysis

SPSS 23.0 software (IBM Corp., Armonk, NY, USA) was used for statistical analysis. Kolmogorov–Smirnov test was used to evaluate normality and the Levene test was used to evaluate homogeneity of variance. The distribution of scores for different sequences was compared using the Kruskal–Wallis H test, followed by pair-wise comparisons using the Bonferroni correction. The objective data were compared using one-way analysis of variance. Mann-Whitney U test was used to compare the score differences with the three diffusion sequences. The consistency of the subjective rating results reported by the two raters was evaluated by the kappa consistency test (kappa value >0.75 indicated high consistency, 0.4–0.75 indicated moderate consistency, and <0.4 indicated low consistency). The intra-class correlation coefficient was calculated based on a two-way mixed model to test the consistency between the two readers. Intra-class Correlation Coefficient (ICC) values of <0.50, 0.50–0.75, 0.75–0.90, and >0.90 represented poor, moderate, good, and excellent degree of agreement between the readers, respectively. The independent t-test was used to compare the differences in mean ADC values of the three diffusion sequences. ROC analysis was performed to evaluate the performance in differentiating benign from malignant prostate diseases. The Delong test was employed to compare the AUC of the three DWI sequences. *p*<0.05 was considered statistically significant.

## 3 RESULTS

### 3.1 Clinical Data

A total of 51 patients were enrolled in this study, which included 31 patients with prostate cancer, 11 patients with prostate benign disease, and 9 patients with bladder cancer. The mean age of the patients was 68±9 years (Table **2**). All patients in this study were confirmed by prostatectomy or biopsy.

### 3.2 Subjective Evaluation

The images acquired using DWI sequences for all 51 patients were clear, and the image quality met the requirements of diagnosis. The subjective rating results of the two readers were of high or moderate consistency (Table **3**). The subjective rating results with respect to the clarity of the images acquired using the three DWI techniques were significantly different, and the post-hoc pair-wise comparisons demonstrated that the scores of clarity between ss-EPI DWI and ZOOMit DWI images, as well as ZOOMit DWI and RESOLVE DWI images, were significantly different (*p*<0.05). However, the subjective clarity score was not significantly different between the ss-EPI DWI and RESOLVE DWI images (*p*>0.05). The subjective scores of distortions of the images were significantly different, and the post-hoc pair-wise comparisons also demonstrated that the distortion scores between the ZOOMit DWI and RESOLVE DWI images were significantly different (*p*<0.05), while the scores between ss-EPI DWI and ZOOMit DWI images, or ss-EPI DWI and RESOLVE DWI images, were not significantly different (*p*>0.05). The subjective scores of artifacts of the images were also significantly different, and the post-hoc pair-wise comparisons demonstrated the scores of artifacts as well as ZOOMit DWI and RESOLVE DWI images to be significantly different (*p*<0.05). The distribution of the subjective scores of the three DWI sequences is given in Figs. (**S1** and **S2**). Box and radar plots of the subjective parameters of different DWI sequences are shown in Figs. (**S1** and **S2**).

### 3.3 Objective Evaluation

The consistency between the two readers regarding the SNR (b = 50), CNR (b = 50), and SNR (b = 1400) of the three DWI sequences was high or moderate (Table **4**). The values of SNR (b = 50) and SNR (b = 1400) significantly differed among the three DWI sequences (*p* < 0.05; Table **4**). In contrast, the CNR values (b = 50) of the three DWI sequences were not significantly different (*p* > 0.05; Table **4**). The AP diameter, RL diameter, ratio of AP/RL diameter, and area of the prostate measured by the two readers were highly or moderately consistent (Table **4**). When using the T2W image as the reference, the AP diameter and prostate area did not significantly differ in the three DWI techniques (*p* > 0.05; Table **4**). However, the RL diameter and ratio of AP/RL were significantly different between ss-EPI and ZOOMit DWI and between ZOOMit DWI and RESOLVE DWI, respectively (*p* < 0.05; Table **4**). Box plots of the objective parameters of different DWI sequences are shown in Fig. (**S3**).

### 3.4 Differentiating Prostate Cancer and Prostate Benign Disease

The consistency of the PI-RADS scores between the two radiologists using kappa was moderate, and the consistency of the ADC values between the two radiologists in differentiating benign and malignant prostate diseases was moderate, good, or excellent (Tables **S1**, **S2**). In 42 patients, PI-RADS scores and ADC values of the three diffusion sequences were compared between benign and malignant groups (Tables **S3** and **S4**). The three diffusion sequences between benign and malignant prostate tumors are shown in Figs. (**S3**-**S7**).

**Fig. (1):**
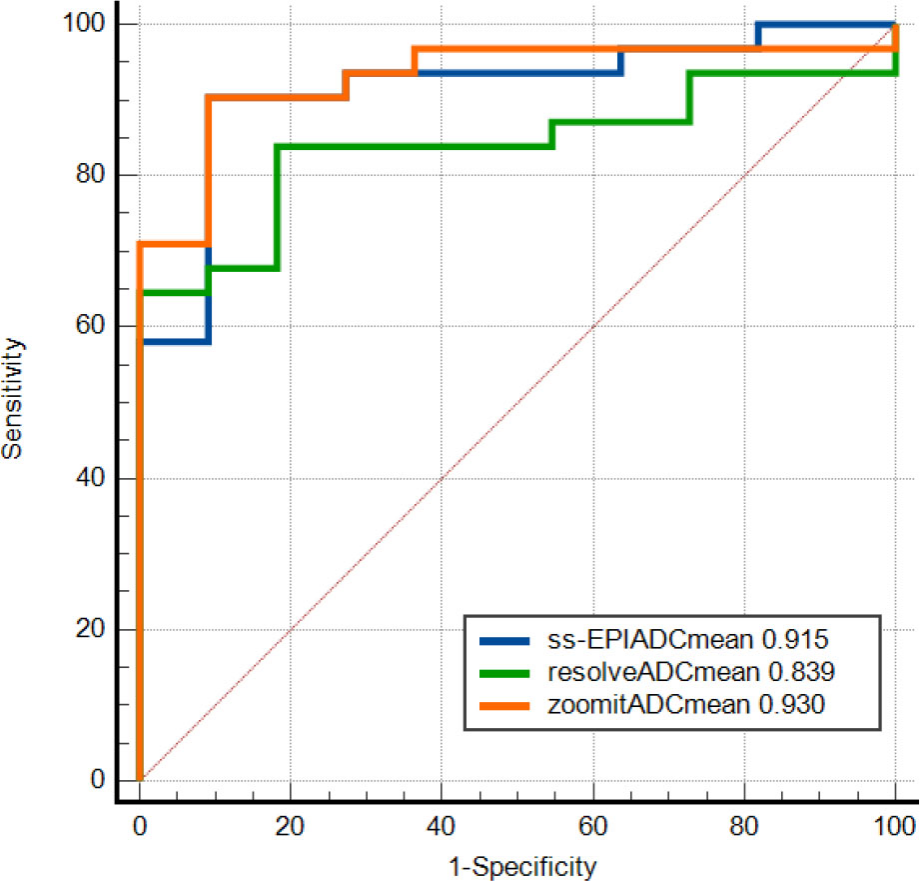
ROC curves of the ADC of the 3 diffusion sequences in benign and malignant prostate diseases (n=42). A comparison of the AUC of any two curves was made by the Delong test (all *p* >0.05).

**Fig. (2):**
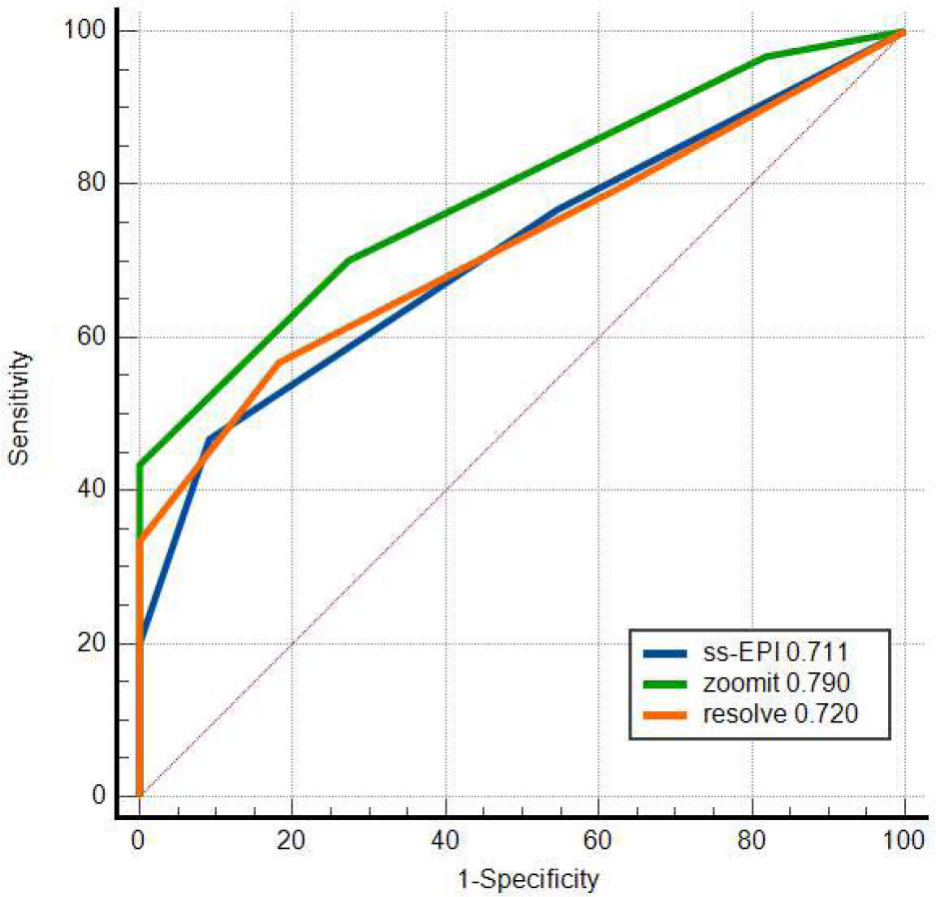
ROC curves of the PI-RADS scores of the 3 diffusion sequences (n=42). A comparison of the AUC of any two curves was made by the Delong test (all *p* >0.05).

Receiver Operating Characteristic (ROC) analysis was used to evaluate the performance of discriminating between malignant and benign prostate disease. The AUC of the PI-RADS score for the three sequences was 0.915 (95% CI, 0.787–0.79), 0.930 (0.807–0.985), and 0.839 (0.693–0.934), differentiating prostate cancer from benign prostate disease, respectively (Fig. **1**). The AUC values of ADC for the three sequences were 0.711 (0.551–0.840), 0.790 (0.637–0.900), and 0.720 (0.558–0.848), respectively (Fig. **2**). After the Delong test, the ROC curves of the three DWI sequences were comparable (all *p*>0.05).

## 4 DISCUSSION

This study aimed to subjectively and objectively compare the image quality among ss-EPI DWI, ZOOMit DWI, and RESOLVE DWI sequences for MRI imaging of the prostate and optimize the strategy for high-quality images. mpMRI of the prostate has become an important tool for the diagnosis of prostate diseases. DWI is the leading sequence for the peripheral zone and the second sequence for the transitional zone; hence, it is the most important MRI sequence for the diagnosis of significant prostate cancer in the PI-RADS scoring system [17, 18]. Therefore, optimizing the quality of DWI images of the prostate is a top priority. Image clarity was found to be higher in the ZOOMit DWI than in the other two DWI sequences. The objective evaluation results showed that the average SNR (b=1400) results of ss-EPI DWI and ZOOMit DWI were not substantially different. ZOOMit DWI applies a slight rotation of the field of excitation and motion compensation using an in-plane registration algorithm and complex averaging, and can be used to improve image sharpness and the estimation accuracy of the ADC maps [19], in agreement with the results of the subjective rating. The CNR did not significantly differ among the three DWI sequences.

According to the subjective evaluation, the image distortion was more evident in the RESOLVE DWI than in the ZOOMit DWI sequences; whereas, the subjective image distortion score was not significantly different between RESOLVE DWI and ss-EPI DWI, as well as ZOOMit DWI and ss-EPI DWI. The quantitative analysis by measuring the diameter of the prostate, of which the standard was set by T2W image, also demonstrated the consistency to be higher in ss-EPI DWI with T2W, while the ratio of AP/RL diameter showed ZOOMit DWI to have higher consistency with T2W. These findings demonstrated that the distortion was not substantial in the prostate images acquired using ZOOMit DWI and ss-EPI DWI sequences, in agreement with the subjective rating results in this study. In addition, the findings reported by Thierfelder *et al*. [9] were also in agreement with the results of this study. The differences in the diameter of the prostate measured in images could be caused by the distortion and deformation of the image of the prostate. Distortion of prostate images is a common issue during pelvic scanning, which could be mainly caused by the adjacent bones and gas in the rectum. However, the ZOOMit DWI technique exhibited a higher spatial resolution, and therefore, the distortion of prostate images was not substantial. Furthermore, in this study, the artifacts were more evident in the images acquired using the RESOLVE DWI sequence than ss-EPI DWI and ZOOMit DWI sequences. However, the artifacts were not significantly different between the ss-EPI DWI and ZOOMit DWI sequences.

In Liney GP’s study [20], only ten normal volunteers were scanned for prostate by different MRI sequences. Image quality was evaluated in terms of daily repeatability with RESOLVE and EPI, performing better than ZOOMit. On the contrary, there were two studies that supported ZOOMit DWI for its differentiating value in patients with varicocele and patients with distal bile duct strictures [21, 22]. In this study, either the AUC of the ADCmean (0.930) or PI-RADS score (0.790) for the ZOOMit DWI sequence was higher than that of RESOLVE DWI and ssEPI DWI sequence in differentiating prostate cancer from benign prostate disease. It was consistent with the ZOOMit image of high resolution and low distortion shown in this study. ZOOMit DWI could be more effective in the diagnosis of patients with prostate disease.

The subjective and objective scores obtained in this study were higher for images acquired using the ZOOMit DWI sequence than ss-EPI DWI and RESOLVE DWI sequences. Both subjective and objective scores also suggested that ZOOMit DWI images could more accurately display the outline of the prostate, and provide accurate information for the diagnosis of prostate diseases, positioning of clinical puncture, and postoperative evaluation of treatment efficacy. The high clarity and low distortion of ZOOMit DWI images could be associated with the smallest voxel size of the ZOOMit DWI sequence. Therefore, ZOOMit DWI could be an important sequence for the multiparametric MRI of the prostate, which can provide accurate information to help in diagnosis and clinical treatment. The ZOOMit DWI sequence has already been demonstrated to be suitable for DWI and DTI in previous studies [9, 11]. Li *et al*. [23] reported that the quality of ZOOMit DWI images was higher than ss-EPI DWI images, being in agreement with the findings of this study. It was also reported that the ZOOMit DWI sequence could improve the image quality of various areas, including the prostate, pancreas, spinal cord, neck, and liver [11, 16, 24-26].

This study involved a few limitations. First, the sample size was relatively small. In our future studies, the sample size will be increased to provide more robust data. Second, the different parameters of TE and TR of the three pulse sequences may have a slight impact on the difference in image quality. Further sample expansion is needed to observe whether the images are within an acceptable range. We only compared single-index dispersion models in our research work. In future studies, we can add multi-index dispersion models, such as DKI and DTI. Another technique, diffusion kurtosis imaging, can also provide more abundant tissue signals and it has shown a great advantage in differentiating malignant prostate diseases [27, 28]. Diffusion Tensor Imaging (DTI) is an MRI technique that can measure not only the diffusion velocity of water molecules, but also the direction of diffusion. Therefore, DTI could more accurately describe the diffusion of water molecules and better predict the subtle changes in microstructures within tissues [29].

## CONCLUSION

In conclusion, the images acquired using the ZOOMit DWI sequence were of better quality. Therefore, the ZOOMit DWI sequence could be preferred as a DWI technique for the MRI of the prostate as high-quality images can provide accurate diagnosis.

## Figures and Tables

**Table 1:** Imaging parameter data.

-	**TR**	**TE**	**TA**	**FOV**	**Matrix**	**Slice**	**Thickness**	**Reconstruction Voxel Size**	**b value**
T2WI	2930	99	2:40	200 × 200	320	25	3	0.6 × 0.6 × 3.0	/
ss-EPI DWI	3900	61	3:09	200 × 200	100	25	3	2.0 × 2.0 × 3.0	0,50,1400
ZOOMit DWI	6300	73	4:39	230 × 125	116	25	3	2.0 × 2.0 × 3.0	0,50,1400
RESOLVE DWI	4510	52	8:09	200 × 200	100	25	3	2.0 × 2.0 × 3.0	0,50,1400

**Table 2:** Clinical features of patients.

**Characteristics**	**All Patients (n=51)**	**Malignant Tumor of Prostate^a^ (n=31)**	**Benign Prostate Diseases^b^ (n=11)**	**Bladder Cancer (n=9)**
Sex	-	Male	Male	Male
Age (mean ± SD)	68±9	70±9	70±9	60±10

**Note:** ^a^including 30 prostate acinar carcinomas and 1 neuroendocrine carcinoma of the prostate. ^b^including 1 acute bacterial prostatitis, 8 prostatic hyperplasia, and 2 normal prostates.

**Table 3:** Kappa consistency of the subjective scores reported by the two radiologists (n = 51).

**Image Quality**	**Subjective Scores**			***p* value**	**Kappa**		
	**ss-EPI**	**ZOOMit**	**RESOLVE**		**ss-EPI**	**ZOOMit**	**RESOLVE**
Clarity	3.14±0.40	3.69±0.47	2.84±0.54	0.000^ac^	0.66	0.74	0.53
Distortion	3.10±0.64	3.37±0.56	2.94±0.54	0.001^c^	0.79	0.85	0.88
Artifacts	3.27±0.78	3.39±0.64	2.96±0.66	0.004^bc^	0.69	0.69	0.83

**Note:** ^a^ *p* < 0.05, post-hoc comparison between ss-EPI and ZOOMit sequences. ^b^*p* < 0.05, post-hoc comparison between ss-EPI and RESOLVE sequences. ^c^*p* < 0.05, post-hoc comparison between ZOOMit and RESOLVE sequences. DWI, Diffusion-weighted imaging; SD, Standard deviation.

**Table 4:** ICC consistency of the objective parameters reported by the two radiologic technicians (n=51).

**Objective Parameters**					***p*-value**	**Intraclass Correlation Coefficient (ICC)^d^**			
**MRI**	**ss-EPI**	**ZOOMit**	**RESOLVE**	**T2W**		**ss-EPI**	**ZOOMit**	**RESOLVE**	**T2W**
SNR-50	3.45±0.93	3.02±0.77^ac^	3.99±0.94^b^	/	0	0.527 (0.414-0.679)	0.697 (0.469-0.827)	0.568 (0.242-0.753)	/
CNR-50	2.28±0.75	5.60±1.92	2.39±0.72	/	0.176	0.727 (0.521-0.844)	0.807 (0.714-0.906)	0.772 (0.601-0.807)	/
SNR-1400	5.91±2.20	5.60±1.92^a^	3.60±1.08	/	0	0.822 (0.688-0.898)	0.893 (0.813-0.939)	0.541 (0.370-0.795)	/
AP	3.76±1.47	3.77±1.50	3.75±1.41	3.67±1.41	0.983	0.885 (0.807-0.933)	0.853 (0.755-0.913)	0.759 (0.613-0.855)	0.922 (0.867-0.955)
RL	4.92±1.32	4.27±1.49^ac^	4.93±1.32	4.81±1.26	0.047	0.855 (0.760-0.915)	0.668 (0.428-0.757)	0.840 (0.736-0.906)	0.845 (0.743-0.909)
Area	16.25±10.83	16.27±10.80	16.42±10.82	15.40±10.09	0.962	0.678 (0.535-0.729)	0.924 (0.871-0.956)	0.916 (0.858-0.951)	0.936 (0.890-0.963)
AP/RL	0.76±0.17	0.90±0.26^ac^	0.75±0.15	0.75±0.16	0	/	/	/	/

**Note: **ADC, Apparent diffusion coefficient; AP, Anterior-posterior diameter; CI, Confidence interval; CNR, Contrast-to-noise ratio; DWI, Diffusion-weighted imaging; ICC, Intraclass correlation coefficient; RL, Right-center diameter; AP/RL, Ratio of anterior-posterior diameter/right-center diameter; SD, Standard deviation; SNR, Signal-to-noise ratio; T2W, T2-weighted. ^a^*p* < 0.05, post-hoc comparison between ss-EPI and ZOOMit sequences. ^b^*p* < 0.05, post-hoc comparison between ss-EPI and RESOLVE sequences. ^c^*p* < 0.05, post-hoc comparison between ZOOMit and RESOLVE sequences. ^d^The two-way mixed model was used for the consistency test. ADC was measured in 10^−6^ mm^2^/s, area in cm^2^, and AP and RL in cm.

## Data Availability

All data generated or analyzed during this study are included in this article and its supplementary information files.

## LIST OF ABBREVIATIONS

ss-EPI: Single-shot echo-planar imaging
SNR: Signal-to-noise ratio
CNR: Contrast-to-noise ratio
ICC: Intraclass correlation coefficient
ROC: Receiver operating characteristic
MpMRI: Multiparametric magnetic resonance imaging
DTI: Diffusion tensor imaging
ADC: Apparent diffusion coefficient
SNR: Signal-to-noise ratio
CNR: Contrast-to-noise ratio
ROIs: Regions of interest
SD: Standard deviation
SI: Signal intensity
RL: Right-left
AP: Anterior-posterior

## STANDARDS OF REPORTING

STROBE guidelines were followed.
