# Supplementary material for: Assessment of Prostate MR Image and Predictive Value for Benign Prostate Disease among Different DWI Sequences
Source: Curr Med Imaging. 2025 Jan 2;21:e15734056329976. doi: 10.2174/0115734056329976241209112720 (PMC12784383; doi:10.2174/0115734056329976241209112720)
Supplement: Supplementary file 1 [file CMIM-21-E15734056329976_SD1.pdf]

1573-4056/25

Send Orders for Reprints to reprints@benthamscience.net

BENTHAM SCIENCE

CrossMark

Current Medical Imaging

Content list available at: https://benthamscience.com/journals/cmir

Supplementary Material

Assessment of Prostate MR Image and Predictive Value for Benign Prostate Disease among Different DWI Sequences

Hanli Dan[1,#], Lu Yang[1,2,#], Yuchuan Tan[1], Yipeng Zhang[1], Yong Tan[1], Jing Zhang[1], Min Li[1], Meng Lin[1] and Jiuquan Zhang[1,*]

[1]*Department of Radiology, Chongqing University Cancer Hospital & Chongqing Cancer Institute & Chongqing Cancer Hospital, Chongqing, 400030, China*
[2]*Key Laboratory for Biorheological Science and Technology of Ministry of Education, State and Local Joint Engineering Laboratory for Vascular Implants, Bioengineering College of Chongqing University, Chongqing, 400030, China*

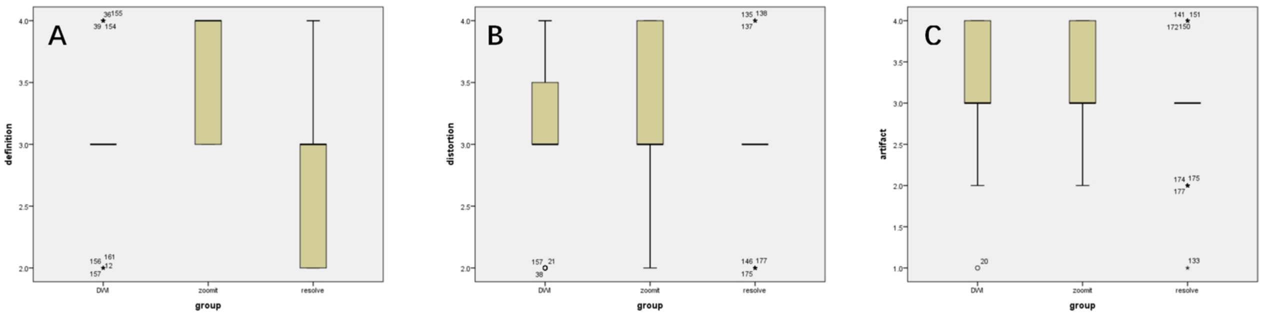

Fig. (S1). Box plot of the subjective scores of different DWI sequences in terms of (**A**), definition, (**B**) distortion, and (**C**) artifact (n=51).

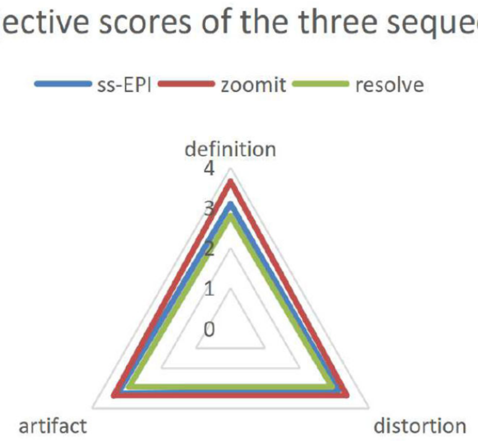

Fig. (S2). Radar plot of subjective scores of the three DWI sequences (n=51).

DOI: 10.2174/0115734056329976241209112720, 2025, 21, e15734056329976

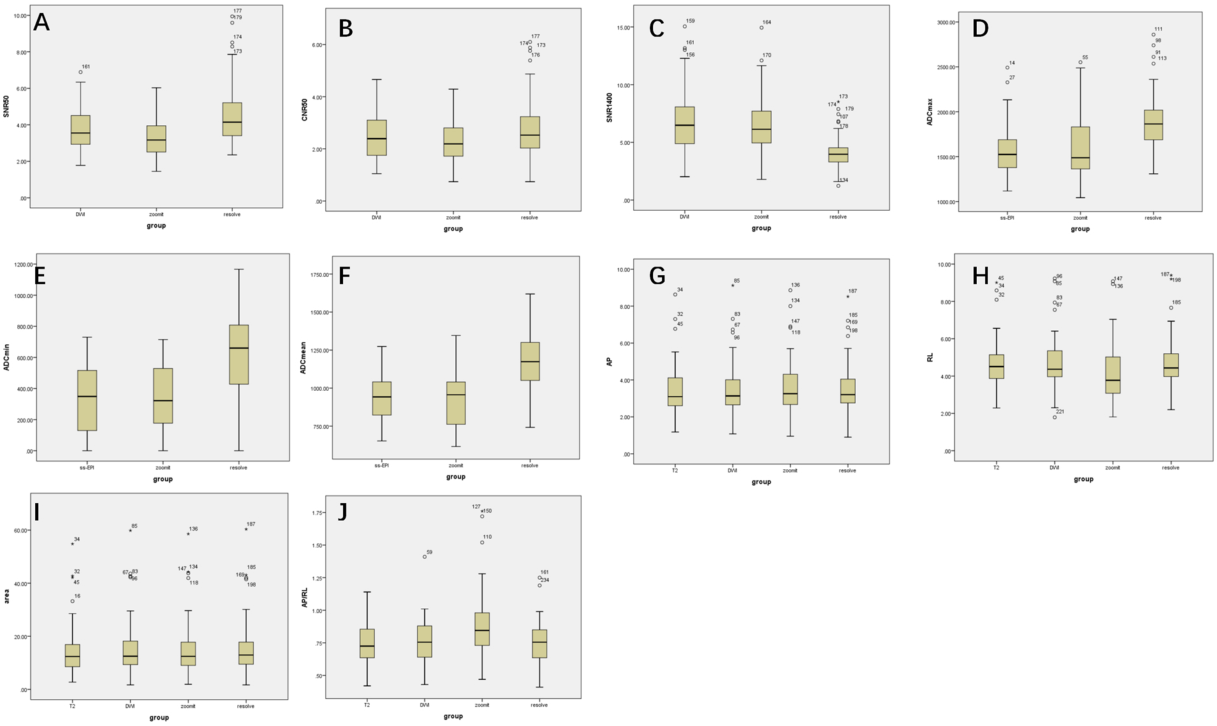

Fig. (S3). Box plot of the objective parameters of different DWI sequences (n=51). (**A**) SNR-50; (**B**) CNR-50; (**C**) SNR-1400; (**D**) ADCmax; (**E**) ADCmin; (**F**) ADCmean; (**G**) AP; (**H**) RL; (**I**) area; (**J**) AP/RL.

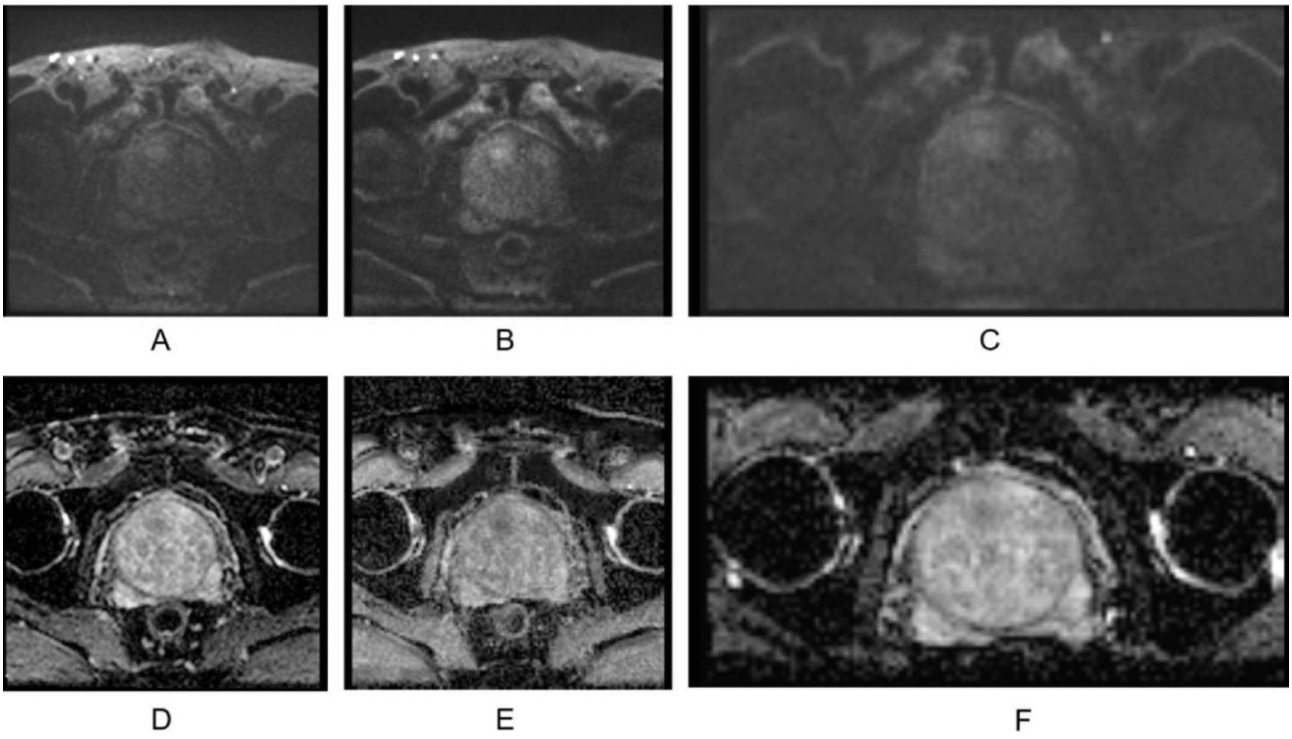

Fig. (S4). A 75 year-old patient with benign prostatic hyperplasia. **A**, **B**, and **C** represent MR ss-EPI, RESOLVE, and ZOOMit images, respectively, showing the prostate diffuse signal to not be uniform, **D**, **E**, and **F** indicate the corresponding ADC images, respectively.

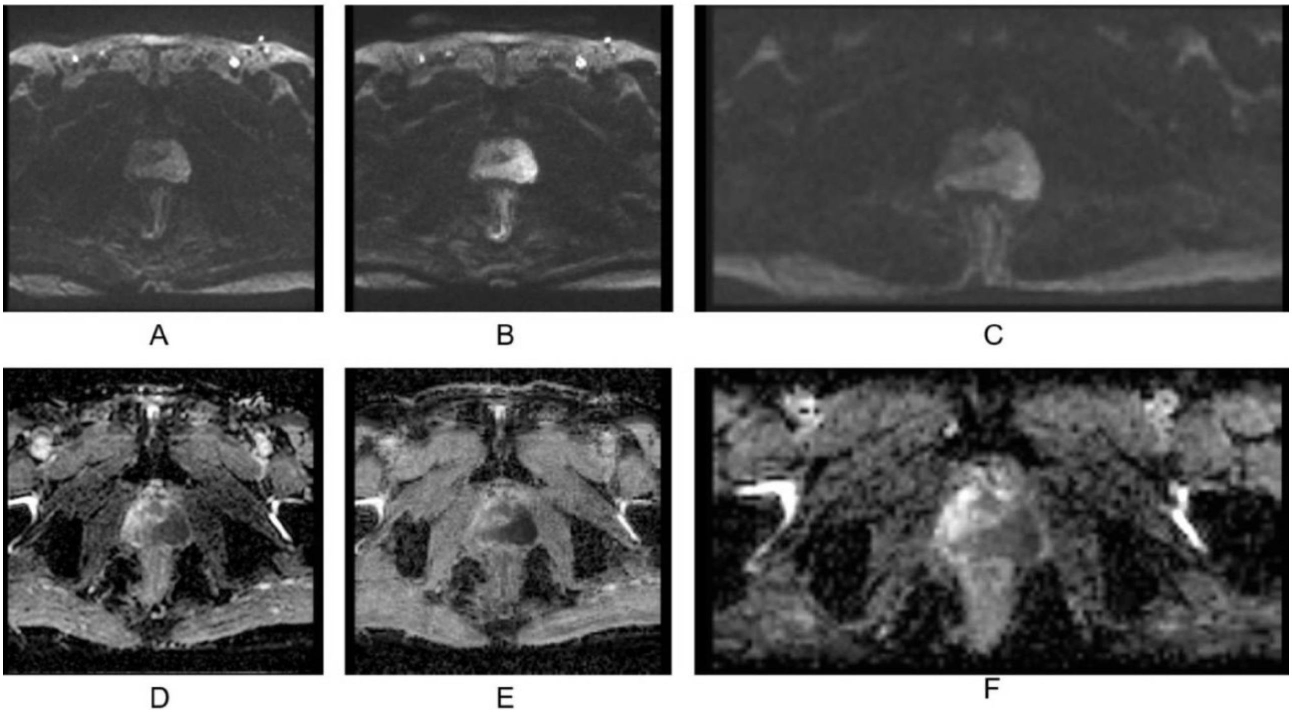

Fig. (S5). A 68 year-old patient with prostate adenocarcinoma. **A**, **B**, and **C** represent MR ss-EPI, RESOLVE, and ZOOMit images, respectively, showing the peri-prostate zone with high signal. **D**, **E**, and **F** indicate the corresponding ADC images, with low signal.

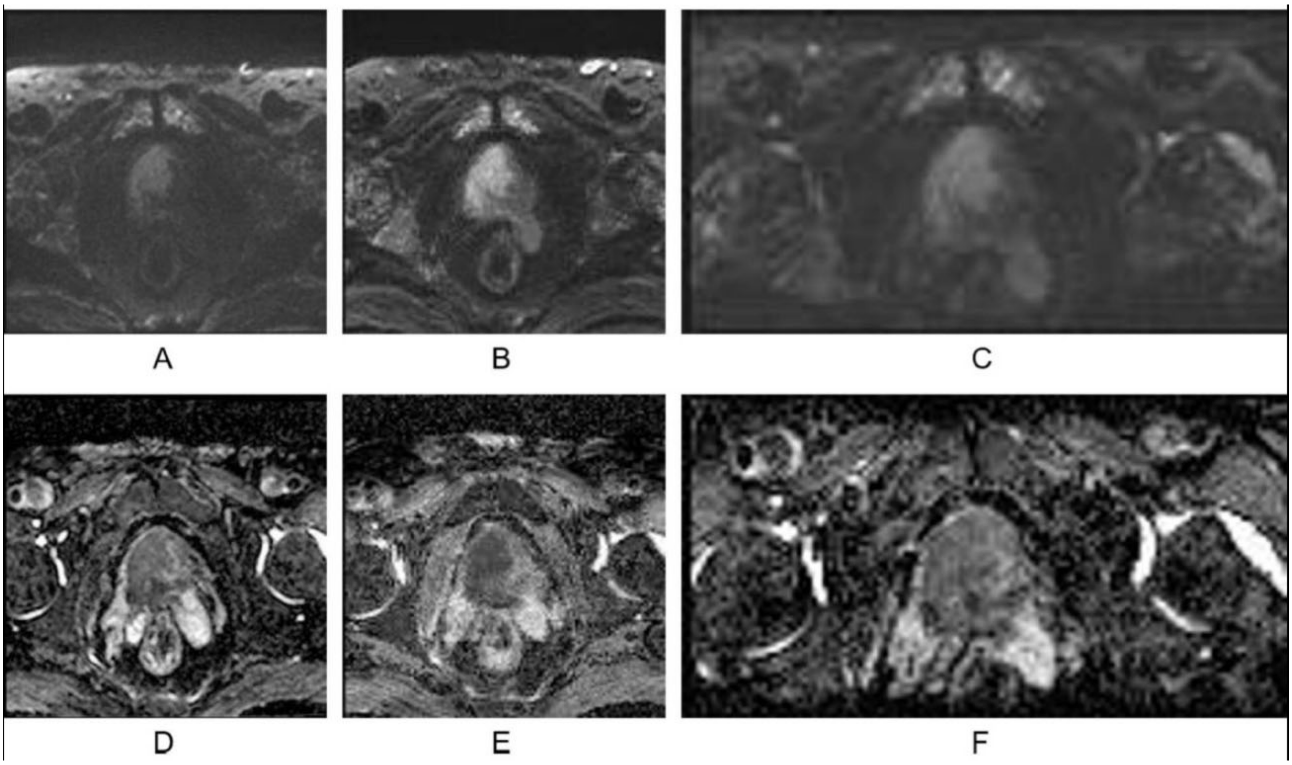

Fig. (S6). A 77 year-old patient with prostate adenocarcinoma. **A**, **B**, and **C** indicate MR ss-EPI, RESOLVE, and ZOOMit images, respectively, showing the right transitional zone and peripheral zone to be high signals. **D**, **E**, and **F** represent the corresponding ADC images with low signals.

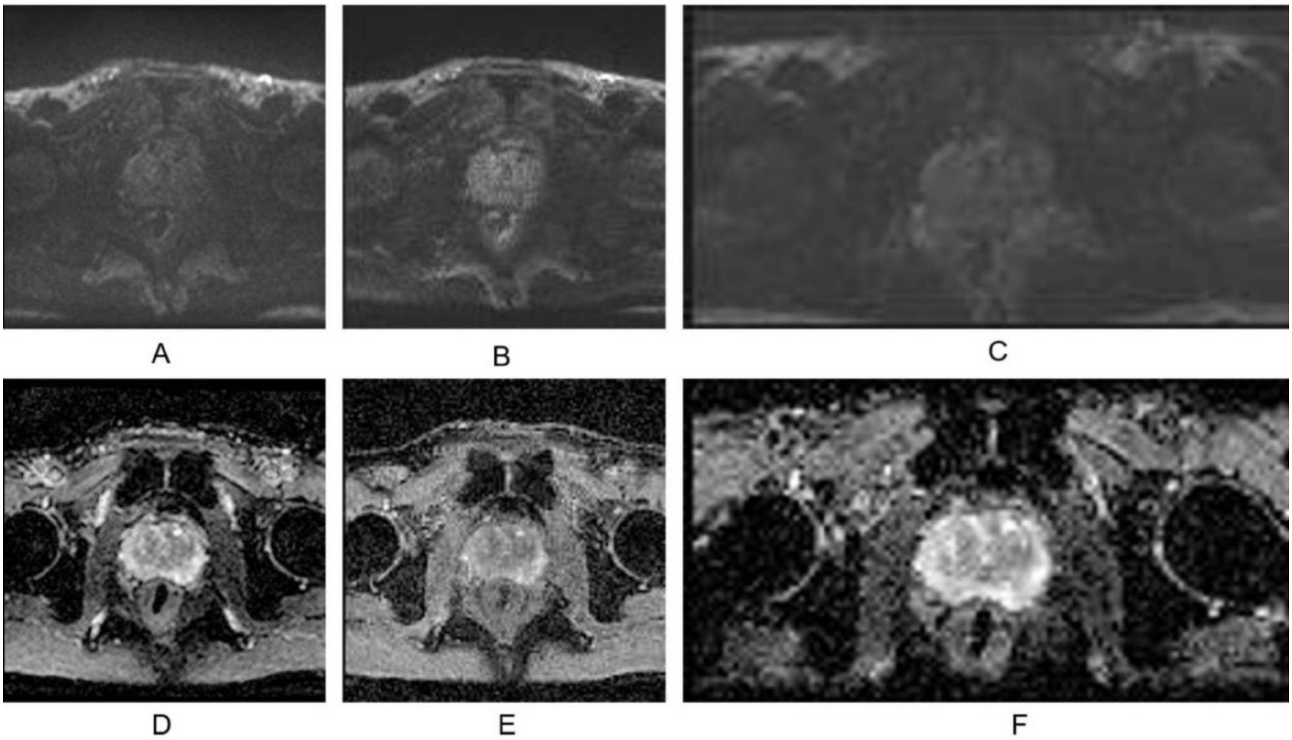

Fig. (S7). A 67 year-old patient with benign prostatic hyperplasia. **A**, **B**, and **C** represent MR ss-EPI, RESOLVE, and ZOOMit images, respectively, showing the prostate diffuse signal to not be uniform, **D**, **E**, and **F** indicate the corresponding ADC images, respectively.

Table S1. Consistency of the PI-RADS scores of the 3 diffusion sequences between the two Radiologists in benign and malignant prostate diseases (n=42).

-	ss-EPI	ZOOMit	RESOLVE
Radiologist 1	3.21±1.025	3.83±0.961	3.46±1.120
Radiologist 2	3.74±1.191	3.86±1.117	3.78±1.194
Kappa	0.686	0.504	0.501
P	<0.001	<0.001	<0.001

Table S2. Consistency of the ADC values of the 3 diffusion sequences between the two radiologists in benign and malignant prostate diseases (n=42).

ADC Parameters	Radiologist 1	Radiologist 2	ICC	95% CI
ss-EPI ADCmax	1584.19±302.46	1482.463±348.76	0.704	0.448-0.841
ss-EPI ADCmin	332.12±221.30	410.64±274.37	0.849	0.719-0.919
ss-EPI ADCmean	936.57±155.32	900.83±236.01	0.876	0.769-0.933
ZOOMit ADCmax	1590.45±363.64	1527.88±408.54	0.84	0.702-0.914
ZOOMit ADCmin	324.14±219.80	416.19±283.61	0.824	0.672-0.905
ZOOMit ADCmean	926.64±175.88	911.82±245.01	0.908	0.830-0.951
RESOLVE ADCmax	1908.79±344.45	1819.36±343.60	0.74	0.516-0.860
RESOLVE ADCmin	599.29±310.90	640.43±302.91	0.863	0.745-0.926
RESOLVE ADCmean	1162.61±191.03	1101.46±251.56	0.838	0.698-0.913

Note: The bi-directional mixed model was used for the ICC consistency test; consistent patterns.

Table S3. Comparison of the PI-RADS scores of the 3 diffusion sequences in benign and malignant prostate diseases (n=42).

DWI Sequences	Malignant, n=31	Benign, n=11	U	P value
ss-EPI DWI	3.42±1.06	2.64±0.67	242.5	0.038
ZOOMit DWI	4.10±0.91	3.09±0.70	269.5	0.004
RESOLVE DWI	3.70±1.15	2.82±0.75	237.5	0.031

Table S4. Comparison of the ADC values of the three diffusion sequences in benign and malignant prostate diseases (n=42).

DWI Sequences	Parameters	Malignant, n=31	Benign, n=11	t	P
ss-EPI DWI	ADCmax	1581.61±337.39	1591.45±183.00	0.092	0.927
	ADCmin	276.87±212.70	487.81±170.09	2.963	0.005
	ADCmean	887.64±144.60	1074.44±88.60	4.007	<0.001
ZOOMit DWI	ADCmax	1539.26±366.09	1734.73±330.25	1.568	0.127
	ADCmin	267.10±207.29	484.91±175.09	3.107	0.003
	ADCmean	865.70±157.70	1098.39±90.85	4.606	<0.001
RESOLVE DWI	ADCmax	1923.03±387.62	1868.64±182.51	0.446	0.658
	ADCmin	576.77±347.48	662.73±167.56	0.784	0.438
	ADCmean	1128.00±208.22	1260.15±73.33	2.046	0.047

© 2025 The Author(s). Published by Bentham Science Publisher.

This is an open access article distributed under the terms of the Creative Commons Attribution 4.0 International Public License (CC-BY 4.0), a copy of which is available at: https://creativecommons.org/licenses/by/4.0/legalcode. This license permits unrestricted use, distribution, and reproduction in any medium, provided the original author and source are credited.
